# Clusters of anatomical disease-burden patterns in ALS: a data-driven approach confirms radiological subtypes

**DOI:** 10.1007/s00415-022-11081-3

**Published:** 2022-03-25

**Authors:** Peter Bede, Aizuri Murad, Jasmin Lope, Orla Hardiman, Kai Ming Chang

**Affiliations:** 1 Computational Neuroimaging Group, Trinity Biomedical Sciences Institute, Trinity College Dublin, Pearse Street, Room 5.43, Dublin 2, Ireland; 2 Department of Electronics and Computer Science, University of Southampton, Southampton, UK

**Keywords:** Amyotrophic lateral sclerosis, Neuroimaging, Biomarkers, Motor neuron disease, Diffusion imaging, Clinical trials

## Abstract

Amyotrophic lateral sclerosis (ALS) is associated with considerable clinical heterogeneity spanning from diverse disability profiles, differences in UMN/LMN involvement, divergent progression rates, to variability in frontotemporal dysfunction. A multitude of classification frameworks and staging systems have been proposed based on clinical and neuropsychological characteristics, but disease subtypes are seldom defined based on anatomical patterns of disease burden without a prior clinical stratification. A prospective research study was conducted with a uniform imaging protocol to ascertain disease subtypes based on preferential cerebral involvement. Fifteen brain regions were systematically evaluated in each participant based on a comprehensive panel of cortical, subcortical and white matter integrity metrics. Using min–max scaled composite regional integrity scores, a two-step cluster analysis was conducted. Two radiological clusters were identified; 35.5% of patients belonging to ‘Cluster 1’ and 64.5% of patients segregating to ‘Cluster 2’. Subjects in Cluster 1 exhibited marked frontotemporal change. Predictor ranking revealed the following hierarchy of anatomical regions in decreasing importance: superior lateral temporal, inferior frontal, superior frontal, parietal, limbic, mesial inferior temporal, peri-Sylvian, subcortical, long association fibres, commissural, occipital, ‘sensory’, ‘motor’, cerebellum, and brainstem. While the majority of imaging studies first stratify patients based on clinical criteria or genetic profiles to describe phenotype- and genotype-associated imaging signatures, a data-driven approach may identify distinct disease subtypes without a priori patient categorisation. Our study illustrates that large radiology datasets may be potentially utilised to uncover disease subtypes associated with unique genetic, clinical or prognostic profiles.

## Introduction

Clinical heterogeneity in ALS is widely recognised. While the diagnosis of ALS requires a core set of clinical features, considerable differences exist in progression rates, disability profiles, survival, cognitive manifestations, and behavioural features [1–4]. Key dimensions of clinical heterogeneity include LMN versus UMN predominance, body region of symptom onset and cognitive profiles, but less characteristic symptoms, such as extrapyramidal, cerebellar, and sensory deficits may also add to the diversity of clinical manifestations [5–8]. The practical upshot of clinical heterogeneity includes the considerable differences in care needs, support services, caregiver burden and resources needed for the multidisciplinary management of the condition. It is widely recognised that individualised supportive strategies are required for the optimal management of ALS, and it is also increasingly accepted the individualised pharmacotherapy may be needed instead of the traditional “one-drug-for-all” approach. The ramifications of disease heterogeneity span beyond patient care and are a considerable challenge in clinical trials which are often hampered by small cohort sizes, stringent entry criteria and high drop-out rates [9]. In line with the concepts of precision medicine, and in recognition of the diversity of clinical trajectories in ALS, a multitude of classification schemes and staging systems were introduced to categorise patient with similar disability, prognostic or cognitive profiles [10–14]. These staging systems are relatively easy to apply in the clinical setting, useful in pharmacological trials, and proved successful in reducing clinical diversity by allocating patients into specific disease categories. Clinical staging, however, require the careful consideration of observed parameters and invariably rely on the interpretation of medical cues, reported symptoms and other potentially subjective factors. An alternative to clinical staging is the exploration of quantitative biomarker data [15–17] to evaluate if distinct subgroups exist, using a data-driven approach relying solely on quantitative, “measured” variables. While the majority of imaging studies use clinical categorisation first to then describe phenotype-, genotype- or stage-associated radiological profiles [18, 19], an alternative is the cluster analysis of pooled imaging data and the subsequent analysis of cluster-associated clinical characteristics. Accordingly, the main objective of this study is the evaluation of a large unsegregated MR dataset with regards to radiological clusters of anatomical involvement without a priori patient categorisation. Our hypothesis is that disease subtypes may be readily identified using a data-driven approach without relying on accompanying clinical variables. A secondary objective of the study is the interrogation of cluster-associated demographic, clinical and genetic information once cluster membership has been established for each participant.

## Methods

### Participants

A total of 214 patients with amyotrophic lateral sclerosis (ALS) were included in a prospective, single-centre study. The study was approved by the institutional ethics board (Beaumont Hospital, Dublin, Ireland), and all participants provided informed consent. Exclusion criteria included prior cerebrovascular events, traumatic brain injury, neurosurgical procedures, as well as comorbid neoplastic, paraneoplastic or neuroinflammatory diagnoses. Participating ALS patients were diagnosed according to the El Escorial criteria. 161 patients were screened for GGGGCC hexanucleotide expansions in *C9orf72*. Methods for genetic screening have been previously reported [20]. GGGGCC repeat expansions in *C9orf72* longer than 30 repeats were considered pathological.

### Magnetic resonance imaging

A standardised imaging protocol was implemented on a 3 Tesla Philips Achieva Magnetic resonance (MR) platform. T1-weighted (T1w) images were acquired with a 3D Inversion Recovery prepared Spoiled Gradient Recalled echo (IR-SPGR) sequence with a spatial resolution of 1 mm^3^, field-of-view (FOV) of 256 × 256 × 160 mm, flip angle = 8°, SENSE factor = 1.5, TR/TE = 8.5/3.9 ms, TI = 1060 ms. Diffusion tensor images (DTI) were acquired with a spin-echo echo planar imaging (SE-EPI) pulse sequence using a 32-direction Stejskal-Tanner diffusion encoding scheme; 60 slices with no interslice gap, spatial resolution = 2.5 mm^3^, FOV = 245 × 245 × 150 mm, TR/TE = 7639/59 ms, SENSE factor = 2.5, *b*-values = 0, 1100 s/mm^2^, dynamic stabilisation and spectral presaturation with inversion recovery (SPIR) fat suppression. To assess for comorbid vascular and neuroinflammatory pathologies, an Inversion Recovery Turbo Spin Echo (IR-TSE) sequence was used to acquire FLAIR images, which were systematically reviewed for each participant. FLAIR data were acquired in axial orientation: spatial resolution = 0.65 × 0.87 × 4 mm, 30 slices with 1 mm gap, FOV = 230 × 183 × 150 mm, TR/TE = 11,000/125 ms, TI = 2800 ms, 120° refocusing pulse, with flow compensation and motion smoothing and a saturation slab covering the neck region.

### Cortical thickness values

The pre-processing of T1-weighted data included non-parametric non-uniform intensity normalisation, affine registration to the MNI305 atlas, intensity normalisation, skull striping, automatic subcortical segmentation, linear volumetric registration, neck removal, tessellation of the grey matter-white matter boundary, surface smoothing, inflation to minimise metric distortion, and automated topology correction [21]. The anatomical labels of the Desikan–Killiany atlas [22] were used to calculate average cortical thickness in the following cortical regions in the left and right cerebral hemispheres separately: (1) banks superior temporal sulcus, (2) caudal anterior cingulate cortex, (3) caudal middle frontal gyrus, (4) cuneus cortex, (5) entorhinal cortex, (6) frontal pole, (7) fusiform gyrus, (8) inferior parietal cortex, (9) inferior temporal gyrus, (10) insula, (11) isthmus–cingulate cortex, (12) lateral occipital cortex, (13) lateral orbitofrontal cortex, (14) lingual gyrus, (15) medial orbital frontal cortex, (16) middle temporal gyrus, (17) parahippocampal gyrus, (18) paracentral lobule, (19) pars opercularis, (20) pars orbitalis, (21) pars triangularis, (22) pericalcarine cortex, (23) postcentral gyrus (24) posterior-cingulate cortex, (25) precentral gyrus, (26) precuneus cortex, (27) rostral anterior cingulate cortex, (28) rostral middle frontal gyrus, (29) superior frontal gyrus, (30) superior parietal cortex, (31) superior temporal gyrus, (32) supramarginal gyrus, (33) temporal pole, and (34) transverse temporal cortex.

### Volume metrics

The brainstem was segmented with a Bayesian parcellation approach into the medulla oblongata, pons, midbrain and superior cerebellar peduncle, based on a probabilistic brainstem atlas derived from 49 scans [23]. A total of 25 volume variables were estimated from each pre-processed T1-weighted dataset: (1) left cerebellar cortex volume, (2) left thalamus volume, (3) left caudate volume, (4) left putamen volume, (5) left pallidum volume, (6) left accumbens volume, (7) left amygdala, (8) left hippocampus, (9) right cerebellar cortex volume, (10) right thalamus volume, (11) right caudate volume, (12) right putamen volume, (13) right pallidum volume, (14) right accumbens volume, (15) right amygdala, (16) right hippocampus, (17) posterior corpus callosum volume, (18) middle corpus callosum volume, (19) central corpus callosum volume, (20) mid-anterior corpus callosum volume, (21) anterior corpus callosum volume, (22) medulla volume, (23) pons volume, (24) superior cerebellar peduncle volume, and (25) midbrain volume, and the total intracranial volume (TIV) was also estimated for each subject. Each volume value was converted as a percentage of the subject’s total intracranial volume (TIV) to account for TIV variations.

### White matter indices

Following quality control, eddy current corrections and skull removal were applied to DTI data before a tensor model was fitted to generate diffusivity maps of fractional anisotropy (FA). FMRIB’s software library’s (v6.0) tract-based statistics (TBSS) module was implemented for the non-linear registration of DTI images, skeletonisation and the creation of a mean FA mask. The study-specific white matter skeleton was masked in MNI space by the anatomical labels of the following white matter regions: left and right anterior thalamic radiation, left and right posterior thalamic radiation, left and right cerebellar white matter skeleton, left and right corticospinal tract, forceps major, body of the corpus callosum, forceps minor, left and right inferior cerebellar peduncle, middle cerebellar peduncle, left and right superior cerebellar peduncle, left and right inferior longitudinal fasciculus, left and right uncinate fasciculus, left and right superior frontal lobe, left and right inferior frontal lobe, left and right temporal lobe, left and right occipital lobe, left and right parietal lobe, left and right cingulum, left and right inferior fronto-occipital fasciculus, left and right superior longitudinal fasciculus, left and right medial lemniscus, fornix, and brainstem. To generate spatial masks for the cerebellar peduncles, medial lemniscus and posterior thalamic radiation, the labels of the ICBM-DTI-81 white matter atlas [24, 25] were used. To create masks for the cingulum, forceps major, forceps minor, body of corpus callosum, anterior thalamic radiation, uncinate, inferior longitudinal fasciculi, superior longitudinal fasciculi, inferior fronto-occipital fasciculi, and corticospinal tracts, the labels of the JHU white matter tractography atlas [26, 27] were utilised. FMRIB’s fornix template [28] was used to mask the study-specific white matter skeleton in MNI space. Labels of the MNI probabilistic atlas [29, 30] was used to generate a white masks for the cerebellum, frontal, temporal, occipital, and parietal lobes. The frontal lobe was divided into inferior and superior sections at MNI coordinate *z* = 8. Label 8 of the Harvard–Oxford probability atlas [31] was used to create a brainstem mask.

### Statistical interpretation

Fifteen key regions of interest (ROIs) were defined covering the entire cerebrum: (1) inferior frontal, (2) superior frontal, (3) peri-Sylvian (lateral sulcus), (4) mesial inferior temporal, (5) superior lateral temporal, (6) parietal, (7) occipital, (8) “motor”, (9) commissural, (10) brainstem, (11) cerebellum, (12) subcortical, (13) limbic, (14) long association fibres, and (15) “sensory”. A total of 25 volumetric variables, 68 cortical thickness values and 40 white matter indices were systematically retrieved from each subject’s imaging data. Integrity metrics of bilateral structures were averaged pairwise, resulting in a total of 74 variables (17 volumes, 34 thickness, 23 FA) in each subject (Table 1). In each anatomical region, cortical thickness values were added and min–max normalised to a 0–1 scale. In subcortical and infratentorial ROIs (corpus callosum, basal ganglia, brainstem, cerebellum etc.), volume values were added and min–max normalised instead. With the exception of the long association ROI, where the two input FA values were 0–1 scaled separately, white matter metrics in each other ROI were added and 0–1 scaled. As a result, in each ROI there were two 0–1 scaled indices which were added for a single composite score representing the integrity of the ROI ranging from 0 to 2, higher scores indicating superior regional integrity, lower scores representing degenerative change.

**Table 1:** Definition of regions of interest and input imaging variables

	**ROI**	**Cortical thickness/volume metrics**	**White matter metrics**
1	Inferior frontal	Lateral orbitofrontal th. Medial orbitofrontal th. Pars orbitalis th. Frontal pole th. Rostral anterior cingulate th.	Inferior frontal FA
2	Superior frontal	Superior frontal th. Rostral middle frontal th. Caudal middle frontal th. Caudal anterior cingulate th.	Superior frontal FA
3	“Peri-Sylvian”	Pars opercularis th. Pars triangularis th. Insula th.	Uncinate fasciculus FA
4	Mesial-inferior temporal	Entorhinal th. Parahippocampal th. Fusiform th. Temporal pole th.	Inferior longitudinal fasciculus FA
5	Superior-lateral temporal	Superior temporal th. Middle temporal th. Inferior temporal th. Transverse temporal th. Banks of the superior temporal sulcus th.	Average temporal FA
6	Parietal	Inferior parietal th. Superior parietal th. Supramarginal th. Precuneus th. Posterior cingulate th. Isthmus cingulate th.	Average parietal FA
7	Occipital	Lateral occipital th. Lingual th. Cuneus th. Pericalcarine th.	Average occipital FA
8	Motor	Precentral th. Paracentral th.	Corticospinal tract FA
9	Commissural	Posterior, middle, central, mid-anterior, anterior corpus callosum vol.	Forceps major FA Forceps minor FA Body of corpus callosum FA
10	Brainstem	Medulla vol., pons vol., midbrain vol	Brainstem FA
11	Cerebellum	Cerebellar cortex vol., superior cerebellar peduncle vol.	Inferior cerebellar peduncle FA, middle cerebellar peduncle FA, superior cerebellar peduncle FA, average cerebellar FA
12	Subcortical	Thalamus vol., caudate vol., putamen vol., pallidum vol., accumbens vol.	Anterior thalamic radiation FA, posterior thalamic radiation FA
13	Limbic	Amygdala vol., hippocampus vol.	Fornix FA Cingulum FA
14	Long association fibres	–	Inferior fronto-occipital fasciculus FA, superior longitudinal fasciculus FA
15	Sensory	Postcentral gyrus	Medial lemniscus FA

*ROI* region-of-interest, *FA* fractional anisotropy, *th.* thickness, *vol.* volume

Based on the 15 regional integrity scores, a 2-step cluster analysis was conducted using Euclidean distance measure. The number of clusters was not fixed a priori, and the Bayesian Information Criterion (BIC) was used to determine the number of clusters. Based on cluster membership of individual patients, cluster sizes were determined and silhouette analyses run using the STATS CLUS SIL extension of SPSS. The hierarchy of input variables was calculated to rank predictor importance, i.e. the measures of which brain regions best segregate the patients. Cluster membership was plotted in a scatter plot along the integrity gradient of the three most relevant ROI to demonstrate case separation. In post hoc analyses, the clinical and genetic profiles of the clusters were contrasted.

## Results

**Fig. 1:**
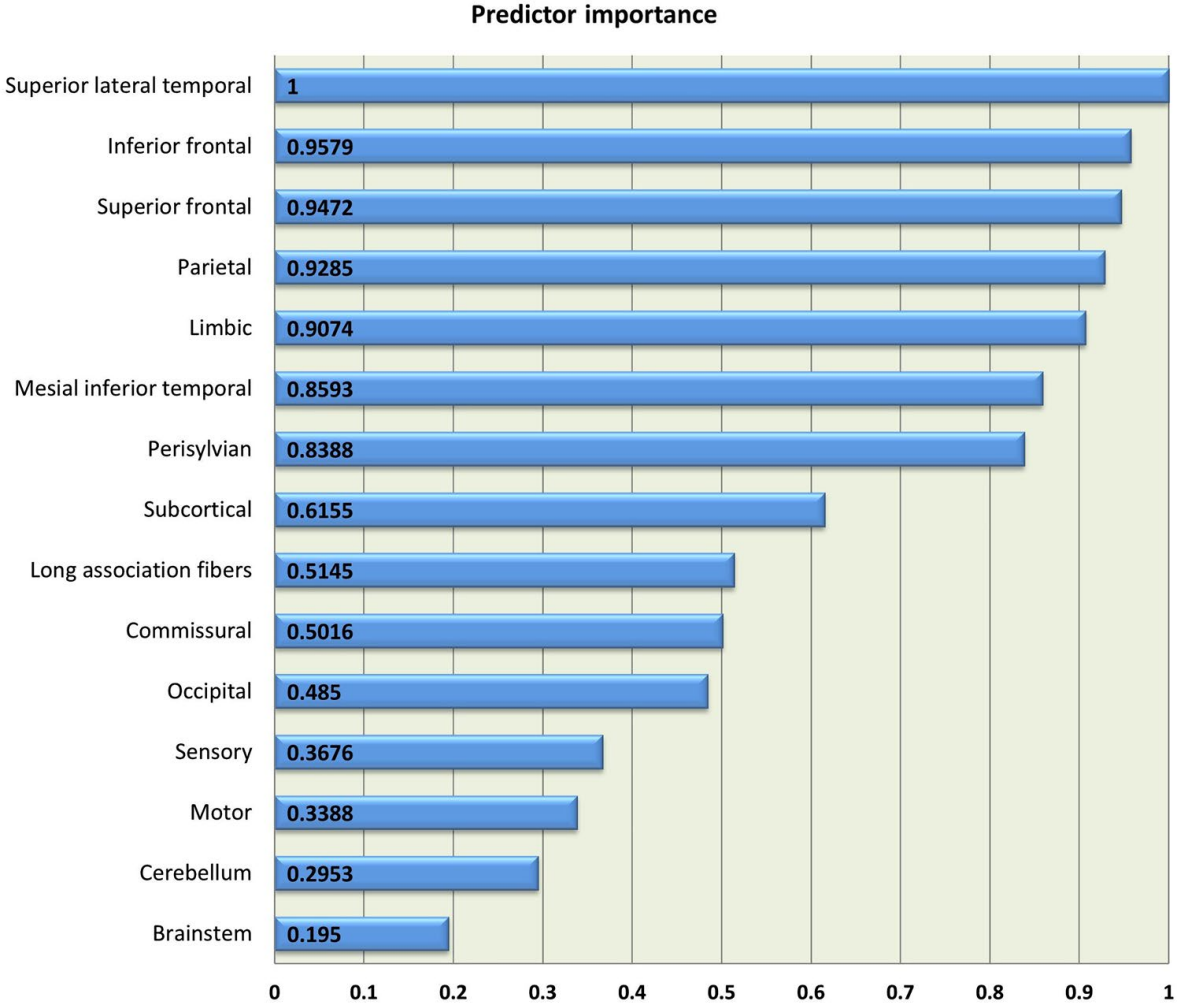
The relative predictor importance profile of each anatomical region

Two-step cluster analysis identified two distinct clusters of anatomical disease-burden distribution, 35.5% of patients (*n* = 76) segregating to Cluster 1 and 64.5% (*n* = 138) to Cluster 2. The silhouette coefficient of 0.572 indicates reasonable cohesion and separation according to Kaufman and Rousseeuw [32]. Variable ranking revealed that the ROIs that best predict cluster membership are superior lateral temporal, inferior frontal, superior frontal, parietal, limbic, mesial inferior temporal, peri-Sylvian, subcortical, long association fibres, commissural, occipital, ‘sensory’, ‘motor’, cerebellum, and brainstem in descending order of importance (Fig. 1).

**Fig. 2:**
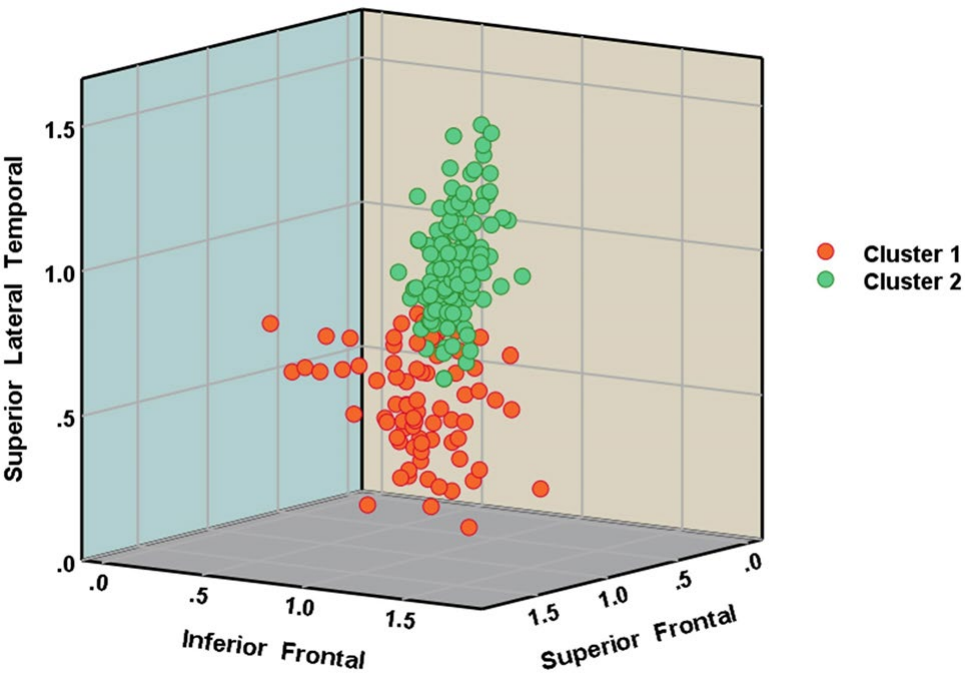
3D scatter plot of patients in Cluster 1 and Cluster 2 based on the integrity of the superior lateral temporal, inferior frontal and parietal ROI

To illustrate the discrimination potential of the anatomical regions between the clusters, a scatter plot was generated based on the integrity of the three most relevant anatomical regions (Fig. 2).

Based on allocated cluster membership, the demographic, clinical and genetic profiles of the two clusters were evaluated; *Cluster 1*: *n* = 76 age: 61.9 ± 11.9, male: 54 (71.1%), right handed: 73 (96.1%), education 13.5 ± 3.2, spinal onset: 67 (88.2%), ALSFRS-r: 37.9 ± 5.4, impaired on ECAS: 20 [26.3% of subjects in the cluster, 29.8% of subjects with ECAS available in the cluster (*n* = 67)], ALS-FTD: 13 (17.1%), *C9orf72* hexanucleotide carriers: 16 (21.1% of cluster).

*Cluster 2*: n = 138, age: 60.5 ± 11.9, male: 86 (62.3%), right handed: 129 (93.5%), education 14.1 ± 3.3, spinal onset: 119 (86.2%), ALSFRS-r: 38.8 ± 6.2, impaired on ECAS: 21 (15.2% of subjects in the cluster, 17.9% of subjects with ECAS available in the cluster (*n* = 117)), ALS-FTD: 15 (10.9%), *C9orf72* hexanucleotide carriers: 6 (4.3% of cluster). The two clusters were matched for age (*p* = 0.39), sex (*χ*^2^_corr._ = 1.29, *p* = 0.256), handedness (*χ*^2^_corr._ = 0.22, *p* = 0.64), education (*p* = 0.25), site of symptom onset (*χ*^2^_corr._ = 0.35, *p* = 0.85), ALSFRS-r (*p* = 0.28), presence of comorbid FTD (*χ2*_corr._ = 1.17, *p* = 0.28), and the proportion of patients with impairment on ECAS (*χ2*_corr._ = 3.99, *p* = 0.136). The two clusters differed in genetic profiles (*χ2*_corr._ = 23.17, *p* < 0.0001). In Cluster 1, there were 16 hexanucleotide repeat carriers which is 23.5% of patients with genetic information available in the cluster (*n* = 68). In Cluster 2, there were only six hexanucleotide repeat carriers which is 6.5% of patients with genetic information available in the cluster (*n* = 93). Of the 22 hexanucleotide carriers included in the study, 72.7% (*n* = 16) clustered to Cluster 1, and only 27.3% (*n* = 6) to Cluster 2.

## Discussion

Our data confirm the radiological clustering of ALS into two relatively distinct subtypes. Contrary to previous staging or classification studies, we have not incorporated any complementary clinical, demographic or genetic information and uncovered two distinct subtypes based on the anatomical distribution of degenerative change alone in a large cohort of pooled ALS patients. The motivation behind our approach was to solely interpret objective, quantitative, spatially coded radiological data without applying any a priori stratification strategy. Whilst most studies first categorise patients based on clinical, genetic or phenotypic criteria to then describe phenotype- or genotype-associated imaging signatures, our intention was the opposite; evaluate the natural segregation of patients based on pathological patterns and then assess clinical features associated with the clusters.

Using cerebral grey and white matte measures in 15 cerebral regions covering the entire brain, we have detected 2 distinct subgroups: a larger cluster (64.5%) of patients with moderate extra-motor disease burden and a smaller cluster (35.5%) with considerable frontotemporal pathology. One of the objectives of the study was to evaluate which brain regions best distinguish the subgroups; therefore, the evaluation of predictor importance is of particular interest. The marked involvement of superior lateral temporal and frontal regions in a subset of patients is consistent with previous reports, but the high predictor importance of parietal changes merits further discussion. ALS is not traditionally associated with preferential parietal atrophy [33]. Parietal changes have been sporadically described mostly in association with advanced disease, but our study suggests that parietal indices may help to segregate patients into subgroups. Predictor importance ranking also revealed that the involvement of brain regions traditionally associated with ALS, such as the motor cortex, corticospinal tracts, commissural structures and brainstem do not readily distinguish disease clusters as the pathology of these regions represent core, unifying features of disease [34, 35]. The low predictor importance of the cerebellum is also of interest. Cerebellar changes in ALS have only been recently characterised in detail [36] and the gravity of cerebellar changes are thought to be associated with specific genotypes and phenotypes [7, 37]. While an ALS-ataxia continuum was proposed by some, our data did not indicate the existence of a cluster of patients with marked cerebellar involvement without frontotemporal change.

The heterogeneity of limbic involvement is consistent with the vast body of neuropsychology and neuroimaging literature [38–40]. The importance of mesial inferior temporal structures in segregating ALS subtypes is consistent with the literature of medial temporal pathology in ALS and their contribution to cognitive deficits [18, 41]. Peri-Sylvian features ranked relatively high in our study, despite the left–right averaging of integrity variables. Peri-Sylvian regions are seldom assessed specifically in ALS, as the focus of imaging studies in ALS-FTD is often orbitofrontal, dorsolateral prefrontal and various temporal regions. Preferential insular and Broca’s area degeneration have been previously described in association with *C9orf72* [42] but also often detected in whole-brain cortical thickness or morphometric analyses. Language deficits in ALS are also relatively well described [43, 44], but rarely linked to focal degenerative change [45]. Subcortical integrity metrics ranked to the middle of predictor hierarchy which is somewhat unexpected given the role of subcortical structures driving neuropsychological manifestations and the notion that hexanucleotide carriers may exhibit particularly marked subcortical degeneration [46–49]. Sensory areas ranked low in their importance of separating the clusters, despite recent reports of subtle or subclinical sensory deficits in ALS [50, 51]. Our predictor analysis outcomes highlight the importance of systematically assessing each brain region in ALS instead of only pursuing the analysis of brain regions which are known to be affected based on post mortem data. Certain anatomical areas such as the parietal lobes and occipital lobe may not be characteristic regions of degeneration, yet, as illustrated, may have a role in segregating specific ALS subtypes. This observation is consistent with the emerging machine-learning literature of ALS [52, 53] which suggests that feature importance analyses, especially in multi-class classification schemes, may identify brain regions which are not classically associated with ALS [54, 55].

Our data indicate a relative discordance between clinical and radiological profiles. While subjects in Cluster 1 exhibited marked frontotemporal change radiologically and the proportion of patients with cognitive impairment was higher, the statistical comparison of clinical variables in the two clusters did not reach significance. Furthermore, the two clusters were also matched in motor disability as indicated by their ALSFRS-r profiles. The dissociation between disease burden and clinical performance is increasingly recognised [56] and a multitude of factors, such as compensatory processes, “motor reserve” and “cognitive reserve” may contribute [57–59]. The relative genetic segregation of subjects based on their imaging profiles is of particular interest; 72.7% of hexanucleotide carriers segregated to Cluster 1, and only 27.3% to Cluster 2. It is conceivable, that in much larger datasets, imaging may have a role in uncovering anatomically unique subgroups which may carry a higher percentage of specific genetic variants or groups with distinct clinical features.

Our study is not without limitations. A silhouette coefficient of 0.572 can only be interpreted as a “reasonable” structure [32]. Our cluster analysis relied on cross-sectional data, and similarly to other studies [53, 60], the clinical implications of cluster membership need to be characterised further with regards to potential prognostic and survival ramifications. The assessment whether radiological cluster membership is consistent longitudinally throughout the course of the disease would be of interest [61]. Furthermore, only very basic clinical variables were appraised in the resulting anatomical clusters such as composite disability scores and cognitive screening outcomes. The fine-grained assessment of specific clinical domains such as pyramidal, extrapyramidal, cerebellar, language, social cognition, and apathy scores may reveal significant inter-cluster differences [4, 5, 62–64]. To explore patient segregation into core pathological patterns, relatively large anatomical regions were defined and only structural integrity metrics evaluated. The incorporation of spinal cord metrics [65, 66] and functional network integrity indices [17, 67] may have helped to identify additional clusters. Only symptomatic patients with an established diagnosis of ALS were included in this study. Given the considerable evidence of brain [44, 68] and spinal cord [69] alterations long before symptom onset, the anatomical clustering of asymptomatic mutation carriers would be of particular interest. Finally, the inclusion of non-ALS MNDs, such as SBMA, SMA, PLS, PPS or PMA in cluster analyses may be of potential interest to evaluate if these subtypes segregate from ALS based on their radiological profiles [70–76]. Notwithstanding these limitations, our study demonstrates that pooled radiology data may be utilised to uncover disease subtypes which may be associated with unique genetic profiles.

## Conclusions

Cluster analysis of imaging data reveals distinct subtypes in ALS without accompanying clinical information. The interrogation of biomarkers by data-driven approaches helps to explore the heterogeneity of neurodegenerative conditions without a priori patient stratification. With the increased availability of large harmonised datasets, similar analyses may expose unique disease subtypes with distinctive clinical, prognostic or genetic traits.
